# Comparison of Denosumab and Bisphosphonates for Sarcopenia in Postmenopausal Osteoporosis

**DOI:** 10.3390/jcm15135010

**Published:** 2026-06-27

**Authors:** Seda Karaaslan Yetemen, Erhan Hocaoglu, Erdinc Erturk

**Affiliations:** 1 Department of Internal Medicine, Faculty of Medicine, Bursa Uludag University, Bursa 16059, Türkiye; 2 Division of Endocrinology and Metabolic Diseases, Department of Internal Medicine, Faculty of Medicine, Bursa Uludag University, Bursa 16059, Türkiye

**Keywords:** postmenopausal osteoporosis, sarcopenia, bisphosphonate, denosumab, bioelectrical impedance

## Abstract

**Background:** The objective of this study was to compare the effects of bisphosphonates and denosumab on sarcopenia-related parameters, contributing to a better understanding of osteosarcopenia management. **Methods:** This cross-sectional, comparative study assessed physical performance, muscle strength, muscle mass, and sarcopenia category in postmenopausal women aged 65 to 80 years with osteoporosis who were receiving either bisphosphonates or denosumab. Additional groups consisted of untreated osteoporotic patients and controls without osteoporosis (*n* = 50 per group). Muscle mass was assessed using bioelectrical impedance analysis, and the classification of sarcopenia was assessed according to the EWGSOP2 criteria. **Results:** Among the osteoporotic groups, the 4 m walking speed, 5-chair stand test, and timed up-and-go performance did not differ significantly, although denosumab-treated patients had numerically shorter completion times. Nondominant handgrip strength was the only grip parameter showing a significant between-group difference (*p* = 0.026), mainly due to higher values in denosumab-treated patients than in untreated osteoporotic patients; dominant and maximum handgrip strength were comparable. Skeletal muscle mass was higher in the denosumab group than in both the bisphosphonate-treated and untreated osteoporosis groups (*p* = 0.045), whereas ALM, ASM, and the ASMI did not differ significantly. No statistically significant between-group differences were observed in SARC-F scores or sarcopenia classifications. Controls had significantly higher odds of sarcopenia absence compared with untreated osteoporotic patients, and treatment duration was not correlated with sarcopenia-related parameters. **Conclusions:** These findings suggest a favorable but limited association between denosumab treatment and selected muscle-related parameters. Prospective longitudinal studies are needed to determine whether anti-osteoporotic therapies influence osteosarcopenia.

## 1. Introduction

The worldwide prevalence of older adults is steadily increasing, creating growing interest in strategies to reduce physical disability, frailty, and mortality [1]. Within muscle–bone interactions, sarcopenia and osteoporosis often coexist, sharing common risk factors and biological pathways [2]. This condition, termed osteosarcopenia, is clinically relevant because it increases fracture risk, impairs balance, and reduces physical performance [3,4]. Recent meta-analytic evidence suggests that osteosarcopenia affects roughly 18–21% of the global population, although prevalence differs across regions, ranging from about 11% in Europe to 16% in North America, 21% in South America, and 21–23% in Asia [3,5]. This heterogeneity underscores the need to investigate osteosarcopenia-related outcomes in specific clinical populations and treatment settings.

Skeletal muscle, bone, and adipose tissues arise from mesenchymal stem cells [6,7]. Bone-derived osteokines, including osteocalcin, sclerostin, Wnt3a, prostaglandin E2, and RANKL, may influence muscle metabolism, contractility, insulin sensitivity, protein synthesis, and myoblast proliferation [8,9,10]. Conversely, exercise-induced myokines affect bone: irisin and FGF-2 promote osteoblast differentiation, and insulin-like growth factor-1 (IGF-1) stimulates bone formation. Interleukin-6 (IL-6) enhances osteoclastogenesis by inducing RANKL secretion [11]. These interactions suggest that pathways targeted by osteoporosis treatments may also be relevant to muscle function and sarcopenia-related outcomes.

This mechanistic framework is particularly relevant when comparing anti-osteoporotic agents with different modes of action. Bisphosphonates suppress osteoclast-mediated bone resorption by binding to hydroxyapatite at active remodeling sites. Conversely, denosumab functions as a fully human monoclonal antibody that suppresses osteoclastogenesis and osteoclast activity by binding to RANKL, a pivotal orchestrator of the RANK/RANKL/OPG axis [12]. Given the biological links between bone remodeling pathways and skeletal muscle function, it is clinically relevant to explore whether different anti-osteoporotic treatment exposures are associated with sarcopenia-related outcomes.

Although various osteoporosis treatments are well-studied, no pharmacological agent has yet been explicitly approved for sarcopenia or osteosarcopenia [4,13]. Moreover, evidence directly comparing commonly used anti-osteoporotic agents, particularly bisphosphonates and denosumab, in relation to physical performance, muscle strength, muscle mass, and sarcopenia category remains limited. This represents an important research gap, as these treatments act through distinct antiresorptive mechanisms and may therefore have different associations with muscle–bone outcomes in older osteoporotic women.

This study assessed sarcopenia-related outcomes by osteoporosis status and anti-osteoporotic treatment exposure in older women. Its novelty lies in comparing denosumab and bisphosphonate exposure regarding European Working Group on Sarcopenia in Older People 2 (EWGSOP2)-defined sarcopenia categories [14] and muscle-related parameters among postmenopausal women with osteoporosis, untreated osteoporotic patients, and non-osteoporotic controls. The primary endpoint was sarcopenia category distribution across groups; secondary endpoints included physical performance, muscle strength and muscle mass. We hypothesized that untreated osteoporotic patients would have a less favorable sarcopenia profile than controls, whereas anti-osteoporotic treatment, particularly denosumab, would be associated with more favorable muscle-related outcomes. We also examined whether these associations persisted after adjustment for age, comorbidity burden, pathological fracture history, body mass index (BMI) and serum 25-hydroxyvitamin D levels.

## 2. Materials and Methods

This single-center, cross-sectional, comparative, observational study was undertaken at a tertiary-care institution from April 2023 to March 2024. Women aged 65–80 years attending outpatient clinics were screened sequentially, and eligible participants were enrolled until each study group contained 50 women. Demographic, clinical, diagnostic, and treatment information was collected at enrollment from medical and prescription records and clinician-administered histories. Participants were assessed at a single time point and classified into four groups according to osteoporosis status and treatment exposure: (I) individuals with postmenopausal osteoporosis receiving bisphosphonates (BisP), (II) individuals with postmenopausal osteoporosis receiving denosumab (Dmab), (III) untreated individuals with postmenopausal osteoporosis (Osteo), and (IV) non-osteoporotic controls (CG).

Ethical approval declarations: The Local Ethics Committee approved the protocol on 7 March 2023 (decision no. 2023-5/8). All procedures conformed to the Declaration of Helsinki. Comprehensive information regarding the study objectives and protocols was detailed to all eligible candidates, from whom written informed consent was formally secured prior to their enrollment.

### 2.1. Participants

The study included female participants aged 65 to 80 years. All groups, except for the control group, were required to have a diagnosis of postmenopausal osteoporosis.

Based on an a priori power estimation performed in G*Power (v3.1.9.7; Heinrich Heine University Düsseldorf, Düsseldorf, Germany), a minimum sample size of 45 patients per group was established as necessary for the Chi-square analysis of EWGSOP2-defined sarcopenia categories (effect size w = 0.30, alpha = 0.05, power = 80%). The corresponding degrees of freedom were based on the number of sarcopenia categories. This medium effect size was selected a priori because directly comparable studies evaluating sarcopenia classifications across these specific osteoporosis treatment groups were limited. To account for potential data attrition, 50 patients were included within each group, resulting in a total sample size of 200 [15,16].

The following exclusion criteria were applied to the study population: (i) inability to mobilize independently; (ii) cognitive dysfunction; (iii) an estimated glomerular filtration rate (eGFR) <30 mL/min; (iv) presence of active malignancy, organ failure, critical illness, or a pacemaker; (v) diseases or use of medications known to affect bone metabolism, such as systemic glucocorticoids (used for >3 months at a dose of 5 mg/day of prednisolone or equivalent), aromatase inhibitors, antiepileptic drugs, long-term heparin therapy, immunosuppressive agents, thiazolidinediones, or anti-osteoporotic drugs outside the study protocol [17]; (vi) uncontrolled diabetes mellitus; (vii) noncompliance with treatment (defined as any deviation from the standardized dosing frequency or dosage as verified through clinical interviews, medical and prescription record reviews covering the six months prior to enrollment); or (viii) incomplete diagnostic or treatment information. Patients receiving stable thyroid hormone replacement therapy were not excluded if they were clinically and biochemically euthyroid [12].

The study cohort comprised 200 participants, equally distributed across four study groups (*n* = 50 per group). Group 1 (BisP; *n* = 50) included patients with postmenopausal osteoporosis who were receiving bisphosphonates, whereas Group 2 (Dmab; *n* = 50) included patients receiving denosumab. A treatment duration of more than 6 months was used as the minimum threshold for defining active treatment exposure in both treatment groups. Group 3 (Osteo; *n* = 50): Patients with postmenopausal osteoporosis who had not received any treatment for osteoporosis. Patients in this group were classified as untreated if they met one of two criteria: (1) newly diagnosed patients who had not yet initiated pharmacological therapy or (2) previously treated patients who had self-discontinued therapy without follow-up for an extended period—defined as more than 2 years without denosumab, ibandronate, risedronate, or alendronate, or more than 3 years without zoledronate. These thresholds were used to minimize the potential residual effects of these agents [18,19]. The duration of osteoporosis diagnosis was defined as the interval (in years) between the first documented osteoporosis diagnosis and study enrollment; for newly diagnosed, treatment-naive patients, it was recorded as 0 years. Group 4 (CG; *n* = 50): Control group consisting of individuals without a diagnosis of osteoporosis and without a history of pathological fracture.

### 2.2. Bone Density, Fracture Risk and History of Pathological Fracture

Participants’ bone mineral density (BMD), T-scores, and Z-scores were measured by Dual-Energy X-ray Absorptiometry (DXA) using a Horizon Wi DXA system (Hologic Inc., Marlborough, MA, USA). A T-score at the femoral neck or lumbar spine of ≤−2.5 was considered diagnostic for osteoporosis. Based on participants’ medical history and physical examination findings, the FRAX^®^ online assessment tool was employed to derive 10-year probability scores for both hip and major osteoporotic fractures [20]. Prior fracture history was assessed from medical records and clinician-administered interviews. In participants with an established diagnosis of osteoporosis, fractures occurring spontaneously or following a fall from standing height or lower were considered osteoporotic fractures [12], Additionally, sarcopenia risk was evaluated using the SARC-F questionnaire [21].

### 2.3. Physical Performance and Strength

Physical performance was assessed using three standardized measures: the 4 m walk test (4MWT), the 5-chair stand test (5-CST), and the timed up-and-go (TUG) test. Test completion times were recorded in seconds with a stopwatch. All procedures followed the recommendations of the consensus [14]. During the 4MWT, participants walked at their habitual pace, and gait speed was subsequently calculated. The 5-CST recorded the time needed to complete five consecutive rises from a standard chair without using the arms for support. The TUG test quantified the time required to stand up, walk 3 m, turn around, return to the chair, and sit down; it was used as an indicator of functional mobility.

Within the established diagnostic framework, muscle strength was evaluated using handgrip testing and the 5-CST, while physical performance measures were used to classify sarcopenia severity. Thresholds for reduced physical performance were based on the consensus criteria [14]. Participants were considered to have low physical performance when either usual gait speed during the 4MWT was ≤0.8 m/s or TUG completion time was ≥20 s. In contrast, a 5-CST duration of >15 s was interpreted as reduced muscle strength rather than impaired physical performance. These thresholds are listed with supporting references from Studenski et al. and Abellan van Kan et al. for gait speed, Cesari et al. for the 5-chair stand test, and Bischoff et al. for the TUG test [14,22,23,24,25].

Additionally, upper extremity strength was quantified via handgrip strength using a calibrated Camry^®^ dynamometer (Zhongshan Camry Electronic Co., Ltd., Zhongshan, China) [14]. Three measurements were obtained from both the dominant and nondominant hands. For each participant, both the mean of all measurements and the maximum value achieved were calculated and recorded. Handgrip strength values below 16 kg were considered low [14].

### 2.4. Muscle Mass Assessment

Body composition, specifically muscle and fat-free mass, was quantified via bioelectrical impedance analysis (BIA) utilizing a multi-frequency TANITA MC-780MA-N analyzer (Tanita Corporation, Tokyo, Japan) [14,26]. The primary indices derived from these measurements included appendicular lean mass (ALM) and appendicular skeletal muscle mass (ASM), which were documented for further analysis [27]. Although DXA is widely regarded as the reference standard for muscle mass assessment, BIA was selected in accordance with the corresponding consensus recommendations for routine clinical settings because of its portability, absence of ionizing radiation, and clinical feasibility [26,28]. The TANITA MC-780 series (Tanita Corporation, Tokyo, Japan) has also been previously evaluated against DXA for appendicular lean mass assessment in older adults [29].

BIA measurements were performed at approximately the same time of day under standardized pre-assessment conditions using a multi-frequency TANITA MC-780MA-N analyzer. Participants were instructed to fast for at least 4 h, avoid strenuous physical activity, alcohol consumption, and large meals before the assessment, maintain their usual hydration status, void their bladder before measurement, and remove shoes, socks, and metal accessories during the scan [30]. ALM, representing the lean soft-tissue mass of the four limbs, was estimated by BIA using the device’s validated prediction equation. ASM was also derived from BIA measurements. Appendicular skeletal muscle index (ASMI) was calculated by dividing the ASM by the square of the patient’s height (kg/m^2^). Calculations were systematically recorded to provide a comprehensive profile of the patients’ appendicular skeletal muscle status. Low muscle mass was defined as ASM < 15 kg or ASMI < 5.5 kg/m^2^ [14].

### 2.5. Sarcopenia Assessment

All participants were evaluated according to the four-step diagnostic algorithm [14]. Sarcopenia was classified into four categories: no sarcopenia, probable sarcopenia, confirmed sarcopenia, or severe sarcopenia.

Case finding was performed using the SARC-F questionnaire, with scores ≥ 4 indicating clinical suspicion of sarcopenia [21]. Participants scoring ≥ 4 points were considered to have a clinical suspicion of sarcopenia and underwent further evaluation.

Muscle strength was initially evaluated using handgrip strength and the chair stand test; low muscle strength was defined as handgrip strength < 16 kg or 5-CST completion time > 15 s and was used to identify “probable” sarcopenia [14]. Muscle quantity was assessed by BIA-derived ASMI, with ASMI < 5.5 kg/m^2^ indicating low muscle mass. Participants with both low muscle strength and low muscle mass were classified as having “confirmed sarcopenia”. Physical performance was evaluated using the 4MWT test and TUG test; gait speed ≤ 0.8 m/s or TUG ≥ 20 s indicated reduced physical performance. Confirmed sarcopenia accompanied by reduced physical performance was classified as “severe sarcopenia” [14].

### 2.6. Statistical Analysis

The Shapiro–Wilk test was used to assess the distribution of continuous variables. Data with a normal distribution are presented as mean ± standard deviation, whereas non-normally distributed variables are reported as median (IQR). For two-group comparisons, either the independent-samples t-test or the Mann–Whitney U test was applied; for comparisons involving more than two groups, ANOVA or the Kruskal–Wallis test was used, as appropriate. Relationships between categorical variables were examined using Pearson’s chi-square test or Fisher-type exact tests, including Fisher’s exact and Fisher–Freeman–Halton tests. Correlation analyses were performed using Pearson’s or Spearman’s correlation coefficients.

The groups were not individually matched for BMI, age, or comorbidity burden. Therefore, multivariable models were constructed to address potential confounding. Sarcopenia-related parameters were assessed using binary logistic regression, while continuous outcomes were compared between groups using one-way ANCOVA, adjusted for age, BMI, Charlson Comorbidity Index, serum vitamin D levels, and prior pathological fracture history. Results of adjusted comparisons are presented as estimated marginal means ± standard error (SE). All data were analyzed using IBM SPSS statistics (version 28.0; IBM Corp., Armonk, NY, USA) with statistical significance set at a two-tailed *p* < 0.05.

## 3. Results

### 3.1. Baseline Characteristics

A total of 200 patients (50 in each group) were evaluated. The median age of the study participants was 70 years (range: 65–80), with no significant difference observed between the groups. Median osteoporosis-diagnosis duration differed significantly across three osteoporotic groups when the full Osteo group was analyzed [BisP: 4.5 years (IQR 2.0–10.5), Dmab: 6.0 years (IQR 2.0–11.0), Osteo: 0.0 years (IQR 0.0–3.75); Kruskal–Wallis *p* < 0.001], reflecting the predominance of newly diagnosed, treatment-naive patients in the Osteo group (34/50, 68%; median 0 years) relative to the 16 patients (32%) with an established, self-discontinued diagnosis. When the analysis was restricted to non-zero values, osteoporosis-diagnosis duration was not significant [BisP: 4.5 (IQR 2.0–10.5), Dmab: 6.0 (IQR 2.0–11.0), Osteo: 6.0 (IQR 4.75–7.50); *p* = 0.521], indicating that the overall difference was driven by the structural zero values of newly diagnosed patients rather than by any meaningful difference in disease chronicity among patients with an established diagnosis. Although a treatment duration of >6 months was used as the minimum threshold for defining active treatment exposure, the actual treatment duration was substantially longer. The median treatment duration was 35.5 months in the BisP group and 24 months in the Dmab group, with no statistically significant difference between the BisP and Dmab groups (*p* = 0.065). For descriptive context, the overall mean treatment duration among treated participants was 42.4 months.

FRAX risk scores showed no significant differences among Osteo, BisP and Dmab groups (major fracture risk: *p* = 0.576; hip fracture risk: *p* = 0.366). The proportion of patients with a history of pathological fractures was comparable across groups (*p* = 0.281). Likewise, BMD values measured by DXA were not significantly different among these groups (*p* = 0.770).

### 3.2. Dynamic Sarcopenia Parameters and Correlations

In the comparative analysis between the Osteo and the CG, the Osteo group performed more poorly across all functional mobility assessments, specifically the 4MWT, 5-CST, and TUG tests (all *p* < 0.001). Similarly, the Osteo group showed significantly diminished strength in dominant, nondominant, and maximum handgrip assessments relative to the CG (all *p* < 0.005). In the analysis of all patients, higher FRAX major fracture risk scores were associated with increased durations of 4MWT and TUG test times, indicating lower performance (r = 0.21, *p* = 0.011; r = 0.22, *p* = 0.006).

### 3.3. Comparison of Dynamic Sarcopenia Tests Within the Groups

**Figure 1:**
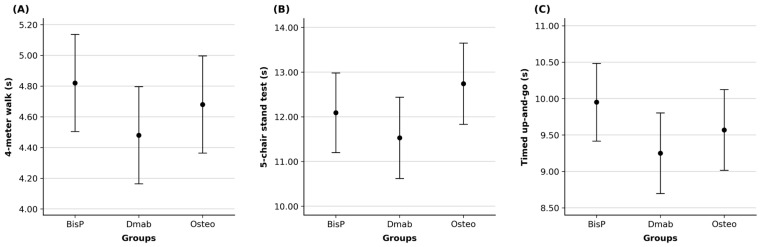
Comparison of dynamic sarcopenia tests among the osteoporosis groups. Confounding factors: Age, BMI, Charlson Comorbidity Index, serum Vitamin D levels and history of pathological fractures. (**A**) 4-m walk test (s), (**B**) 5-chair stand test (s), (**C**) Timed up-and-go test (s). BisP: patients undergoing bisphosphonate treatment, Dmab: patients undergoing denosumab treatment, Osteo: patients who had not received any treatment for osteoporosis.

When the BisP, the Dmab, and the Osteo group were compared, no significant differences were observed in the 4MWT or the 5-CST (*p* = 0.099 and *p* = 0.198, respectively). The Dmab group demonstrated significantly shorter TUG test completion times compared with both the BisP and Osteo groups (*p* = 0.03). No significant difference was found between the Osteo and BisP groups. After accounting for predefined confounders, no statistically significant differences were observed among the three groups in the 4MWT, 5-CST, or TUG test, although the Dmab group completed all of the tests in numerically shorter times. The *p*-values were 0.340, 0.202, and 0.196, respectively (Table 1, Figure 1).

**Figure 2:**
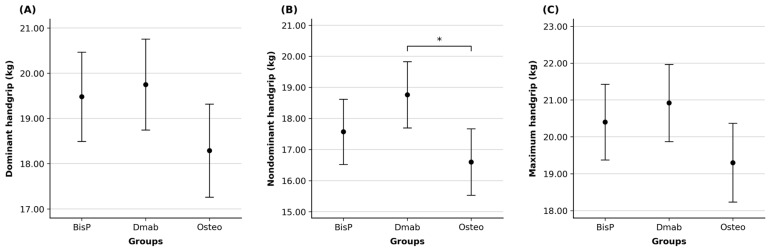
Comparison of handgrip tests among the three osteoporosis groups. Confounding factors: Age, BMI, Charlson Comorbidity Index, serum Vitamin D levels and history of pathological fractures. * *p* < 0.05 for the adjusted pairwise comparison between the Dmab and Osteo groups. (**A**) 4-m walk test (s), (**B**) 5-chair stand test (s), (**C**) Timed up-and-go test (s). BisP: patients undergoing bisphosphonate treatment, Dmab: patients undergoing denosumab treatment, Osteo: patients who had not received any treatment for osteoporosis.

Regarding handgrip strength, there were no significant differences among the Osteo, BisP and Dmab groups in dominant hand, nondominant hand, or maximum grip strength measurements. Following consideration of the previously described confounding factors, no statistically significant differences were observed among these groups in dominant handgrip strength or maximum handgrip strength. However, a significant difference was detected among the three groups in nondominant handgrip strength (*p* = 0.026). Subgroup analyses indicated that this difference was primarily attributable to the difference between the Dmab and Osteo groups (Table 1, Figure 2). Among treated patients (Dmab and BisP groups), treatment duration showed no significant correlations with any dynamic sarcopenia parameters.

### 3.4. Muscle Mass Parameters and Correlations

Muscle mass parameters showed that ALM, SMM and ASM were significantly higher in CG than in Osteo (*p* = 0.003, *p* < 0.001, and *p* = 0.003, respectively). No significant difference was observed in the ASMI (Control group: 7.60 ± 0.65 vs. Osteo: 7.41 ± 0.77, *p* = 0.065). Although ASMI values followed a similar downward trend in the Osteo group, the difference did not reach statistical significance.

When a total of 100 patients from the Osteo and CG were evaluated, lumbar total BMD and femoral neck BMD were found to be positively correlated with ALM, skeletal muscle mass, ASM, and ASMI values (all *p* < 0.05, Table 2).

### 3.5. Comparison of Muscle Mass Parameters Within the Osteoporosis Groups

**Figure 3:**
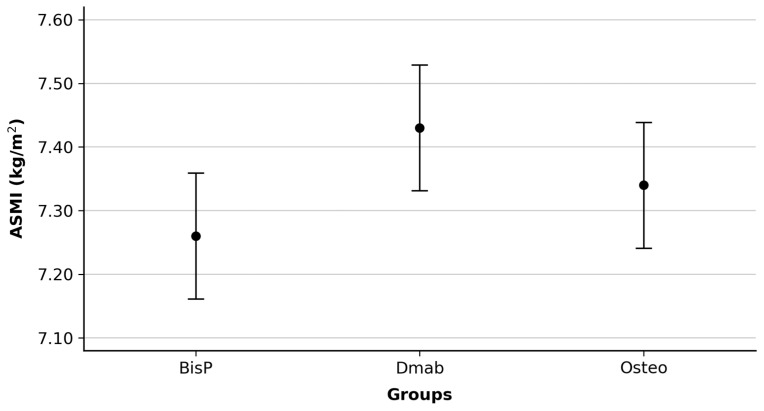
Comparison of ASMI among the postmenopausal osteoporosis groups. ASMI: Appendicular skeletal mass index, BisP: patients undergoing bisphosphonate treatment, Dmab: patients undergoing denosumab treatment, Osteo: patients who had not received any treatment for osteoporosis. Confounding factors: Age, BMI, Charlson Comorbidity Index, serum Vitamin D levels and history of pathological fractures.

Muscle mass parameters were compared across the three osteoporosis groups (BisP, Dmab, and Osteo) (Table 3). Although no significant difference in skeletal muscle mass was observed among the three groups in the crude analyses, a multivariable linear regression model including the predefined confounders showed that both the BisP (β = −0.895 kg, *p* = 0.032) and the Osteo (β = −0.931 kg, *p* = 0.030) had significantly lower skeletal muscle mass compared with Dmab (R^2^ = 0.444, Table 3). ALM and ASM values did not differ significantly among the groups (Table 3). Although ASMI values were numerically higher in the Dmab group, no statistically significant difference was observed among the three groups (*p* = 0.075; Figure 3).

### 3.6. SARC-F Assessment and Sarcopenia Classification

SARC-F scores were significantly higher in the Osteo group than in the CG (*p* < 0.001), whereas no significant difference was observed among the three osteoporosis groups (*p* = 0.112).

Probable, confirmed, or severe sarcopenia were evaluated across the groups (Table 4) [14]. The CG had significantly higher odds of sarcopenia absence than Osteo patients (adjusted OR = 12.99, 95% CI: 2.76–61.09; *p* = 0.001).

Crude analyses demonstrated significant overall group differences in probable sarcopenia and absence of sarcopenia among the three osteoporotic groups (*p* = 0.011 and *p* = 0.049, respectively, Table 4); however, these differences were no longer significant after adjustment for confounders (adjusted *p* = 0.107 and *p* = 0.114, respectively). In pairwise analyses using the Osteo group as the reference, neither bisphosphonate nor denosumab was significantly associated with probable sarcopenia [OR = 0.87, 95% CI: 0.32–2.35, *p* = 0.780; and OR = 0.28, 95% CI: 0.08–1.05, *p* = 0.059, respectively]. For absence of sarcopenia, bisphosphonate was not significantly different from the Osteo group [OR = 1.19, 95% CI: 0.48–2.94, *p* = 0.704], whereas denosumab showed a borderline trend toward higher odds of absence of sarcopenia [OR = 2.89, 95% CI: 0.97–8.56, *p* = 0.056].

## 4. Discussion

Although high-quality evidence supports the efficacy and safety of osteoporosis treatments, research specifically addressing osteosarcopenia remains limited, and no pharmacological agent has yet been approved for its management [4,13]. Some studies have explored the effects of osteoporosis therapies on muscle strength and function in patients with osteosarcopenia; however, investigations incorporating quantitative assessments of muscle mass are scarce [31,32,33,34].

The RANKL/OPG pathway offers a biologically plausible framework for interpreting the selected favorable muscle-related findings observed in the denosumab-treated group. Preclinical evidence indicates that OPG has protective effects on skeletal muscle, whereas OPG deficiency is associated with muscle degeneration and systemic OPG administration improves muscle strength and histological abnormalities in dystrophic models [31,35]. Moreover, pharmacological RANKL inhibition has been shown to improve limb strength, muscle mass, insulin sensitivity, and glucose uptake in experimental models of RANKL overexpression [31]. These data suggest that RANKL inhibition may contribute not only to bone remodeling but also to muscle homeostasis, supporting its relevance in osteosarcopenia.

In line with the recognized bone–muscle interaction in osteosarcopenia, modulation of this pathway may represent a shared mechanism linking bone and muscle metabolism [3]. In this study, our findings support a potential association between RANKL inhibition and selected muscle-related parameters but should be regarded as hypothesis-generating due to the cross-sectional design and lack of longitudinal treatment-response data.

The first notable finding of our study was consistent with previous reports [36,37,38,39], the Osteo group performed poorer on dynamic sarcopenia test results and handgrip strength measures. In addition, muscle mass was significantly lower in the Osteo than in CG. These observations align with earlier studies and further support the link between osteoporosis and reduced muscle mass [40,41]. Together, these results support the validity of subsequent comparisons across the treatment groups.

In our study, no statistically significant differences were observed among the osteoporosis groups in the 4MWT, 5-CST, or TUG test. Nevertheless, the Dmab group completed all dynamic performance tests in numerically shorter times. This pattern suggests that although anti-osteoporotic treatment, particularly denosumab, may be associated with a tendency toward better physical performance, this effect may not be strong enough to remain statistically significant after controlling for major clinical confounders. These findings also indicate that dynamic sarcopenia-related outcomes are likely influenced by multiple factors beyond antiresorptive treatment alone, including age, comorbidity burden, vitamin D status, fracture history, and baseline functional reserve. Therefore, our findings support a cautious interpretation.

In the literature, studies investigating the effects of bisphosphonate therapy on sarcopenia have reported conflicting results. Zhou et al. [42] administered alendronate or placebo to two separate patient groups for 18 months and found no differences in walking speed, TUG, or chair-rise performance between the groups. In contrast, Schacht and Ringe [43] evaluated osteoporotic patients after 3 months of alendronate therapy in a single-arm study and reported significant improvements in physical performance. These discrepancies may be explained by differences in study design, treatment duration, baseline functional status, and whether confounding factors were adequately controlled.

Similarly, the potential functional effects of denosumab remain uncertain. Miedany et al. [44] demonstrated that five-year treatment with denosumab or alendronate, and three-year treatment with zoledronate, led to significant improvements in dynamic tests. The clinical benefits were more notable in patients receiving denosumab than in those on alendronate or zoledronate regimens. In a 6-month prospective study by Phu et al. [45], both denosumab and zoledronate shortened 4 m walk times, and patients treated with denosumab also demonstrated greater improvement in TUG performance. In contrast, Rupp et al. [46] reported no differences between denosumab- and bisphosphonate-treated groups in 5-chair stand test performance following therapy in a retrospective cohort study. Taken together, these findings suggest that denosumab may have a possible favorable effect on physical performance, but the evidence remains heterogeneous.

In our cohort, denosumab-treated patients had the highest absolute handgrip strength values; however, dominant and maximum grip strength did not differ significantly among the three groups. In contrast, nondominant grip strength showed a significant between-group difference (*p* = 0.026), mainly driven by the comparison between the Dmab and Osteo groups. The isolated significance in nondominant handgrip strength may suggest a possible treatment-related trend, but the absence of consistent differences in dominant and maximum handgrip strength precludes a definitive conclusion regarding an independent effect of denosumab on muscle strength. Because the analysis was based on independent group comparisons rather than longitudinal within-person changes, these findings require cautious interpretation.

Studies investigating the effects of bisphosphonate therapy on muscle strength have yielded conflicting results. Zhou et al. [42] reported no change in maximal grip strength after 18 months of alendronate versus placebo in patients over 80 years of age. Similarly, Bahşi et al. [47] found no improvement following zoledronate in geriatric patients. In contrast, Park et al. [48] observed significant increases in grip strength for both hands after 6 months of alendronate plus calcitriol. These conflicting results may be related to differences in patient age, baseline frailty, vitamin D supplementation, treatment duration, and whether muscle strength was assessed longitudinally or cross-sectionally.

Comparative studies of denosumab and bisphosphonates have also yielded heterogeneous results. Miedany et al. [44] demonstrated greater improvements in maximal grip strength with denosumab than with bisphosphonates, whereas Rupp et al. [46] observed significant improvements from baseline in both groups following treatment, without a significant difference between the groups. In a prospective observational study, Bonnet et al. [31] reported a significant increase in maximal grip strength with denosumab, but this increase was not seen in the bisphosphonate and placebo groups. The gain in the denosumab group was strongly correlated with lumbar BMD. These findings suggest that denosumab may confer additional musculoskeletal benefits beyond its established skeletal effects, though the heterogeneity across study designs precludes definitive conclusions [31,44,46].

We assessed muscle mass using BIA, a method supported by sarcopenia guidelines and shown to yield results comparable to DXA [14,26,41]. When comparing osteoporotic groups in our study, both BisP and Osteo had significantly lower skeletal muscle mass compared with Dmab. ALM, ASM and ASMI values did not differ significantly among the groups, although these values were numerically higher in the Dmab group. Prior studies on bisphosphonates have reported inconsistent findings: some observed no change in ALM or the ASMI [33,49,50], while others reported improvements, particularly after longer follow-up with zoledronate [32]. Evidence on the relationship between denosumab therapy and muscle mass remains scarce. Bonnet et al. [31] found a significant increase in ALM among participants treated with denosumab, whereas no comparable increase was observed with bisphosphonate therapy or placebo. In a separate study, Pizzonia et al. [51] reported significant within-group improvements in muscle strength measures in both the denosumab and alendronate groups, but the magnitude of improvement did not differ significantly between treatments. In a recent prospective trial, fat-free mass (FFM) gain after three years of denosumab was greater than with zoledronic acid [34]. Overall, current evidence remains insufficient to determine the clinical effects of denosumab or bisphosphonates on muscle mass parameters.

To the best of our knowledge, this is one of the first studies to compare denosumab- and bisphosphonate-treated groups regarding the prevalence and distribution of probable, confirmed, and severe sarcopenia as defined by the EWGSOP2 criteria [14]. The main contribution of this study is that it evaluates sarcopenia not only as a binary outcome but also through its major components, including muscle strength, muscle mass, physical performance, and sarcopenia categories, within a clinically relevant osteoporotic population.

Among the osteoporotic groups, denosumab was associated with selected favorable muscle-related findings, particularly higher skeletal muscle mass compared with both BisP and Osteo patients, and higher nondominant handgrip strength compared with Osteo patients. However, other parameters and sarcopenia classifications did not differ significantly among osteoporotic groups. Therefore, the novelty of our findings lies not in demonstrating a broad anti-sarcopenic effect of denosumab, but in identifying a selective association between denosumab exposure and specific muscle-related parameters after accounting for relevant confounders.

Study limitations include a small participant pool and a cross-sectional framework, both of which were predicated on the low prevalence of qualified candidates and the unavailability of prior longitudinal muscle metrics for retrospective comparison—alongside unassessed confounding from lifestyle habits. Although diagnosis duration differed across the osteoporotic groups, restricting the analysis to participants with an established osteoporosis diagnosis revealed no significant difference between the Osteo group and either treatment group. This finding supports the stability of the primary comparisons. Although the eligibility criterion required a minimum treatment duration of >6 months, the actual treatment exposure in the treated groups was considerably longer, with an overall mean duration of 42.4 months. Nevertheless, treatment duration may still influence sarcopenia-related outcomes and should be considered when interpreting the findings. The inclusion of self-discontinued patients in the Osteo group reflects real-world practice; pharmacologically defined washout thresholds were applied to minimize residual drug effects [18,19,52].

Overall, this study provides a clinically relevant comparison of sarcopenia-related outcomes across different osteoporosis treatment exposures using standardized sarcopenia classification. In light of the cross-sectional study design, the observed associations between treatment exposure and sarcopenia-related outcomes should not be interpreted as evidence of causality. Given that no pharmacological therapy has yet been approved for osteosarcopenia, randomized prospective trials are needed to clarify the impact of denosumab and bisphosphonates on muscle mass and function. Future investigations should incorporate longitudinal evaluations, standardized sarcopenia criteria, and rigorously account for important determinants, including physical activity and nutritional status.

## 5. Conclusions

In conclusion, this study provides a sarcopenia-oriented comparative evaluation of denosumab, bisphosphonate therapy, and untreated osteoporosis, suggesting selected favorable muscle-related findings in the denosumab group, including higher skeletal muscle mass and higher nondominant handgrip strength. Although these findings do not establish a definitive treatment-specific effect, they suggest that denosumab may be associated with beneficial muscle-related outcomes and support the need for large-scale, randomized, controlled prospective studies to clarify its potential role in the bone–muscle axis.

## Figures and Tables

**Table 1:** Comparison of dynamic sarcopenia and handgrip strength parameters among the osteoporosis groups.

Test	BisP (*n* = 50)	Dmab (*n* = 50)	Osteo (*n* = 50)	*p* ^†^
4 m walk (s)	4.82 ± 0.16	4.48 ± 0.16	4.68 ± 0.16	0.340
5-chair stand test (s)	12.09 ± 0.45	11.53 ± 0.46	12.74 ± 0.46	0.202
Timed up-and-go (s)	9.95 ± 0.27	9.25 ± 0.28	9.57 ± 0.28	0.196
Dominant handgrip (kg)	19.48 ± 0.50	19.75 ± 0.51	18.29 ± 0.52	0.119
Nondominant handgrip (kg)	17.57 ± 0.53 *^ab^*	18.76 ± 0.54 *^a^*	16.60 ± 0.54 *^b^*	**0.026**
Maximum handgrip (kg)	20.40 ± 0.52	20.92 ± 0.53	19.30 ± 0.54	0.108

Data are presented as mean ± standard error (SE). ^†^: One-way analysis of covariance. Bold formatting was used to highlight statistically significant *p*-values (*p* < 0.05). Confounding factors: Age, BMI, Charlson Comorbidity Index, serum Vitamin D levels and history of pathological fractures. *a* and *b* superscripts indicate differences between groups within each row. There are no statistically significant differences between groups with the same superscripts. BisP: patients undergoing bisphosphonate treatment, Dmab: patients undergoing denosumab treatment, Osteo: patients who had not received any treatment for osteoporosis.

**Table 2:** Correlation of bioelectrical impedance analysis (BIA) and DXA results in 100 patients from the untreated osteoporosis (Osteo) group and the control group (CG).

Parameter	Lumbar Total BMD (g/cm^2^)		Femoral Neck BMD (g/cm^2^)	
	*r_s_*	*p*	*r_s_*	*p*
ALM (kg)	0.31	0.020	0.35	**<0.001**
SMM (kg)	0.38	<0.001	0.44	**<0.001**
ASM (kg)	0.31	0.002	0.34	**0.001**
ASMI (kg/m^2^)	0.29	0.004	0.25	**0.012**

ALM: Appendicular lean mass, ASM: Appendicular skeletal mass, ASMI: Appendicular skeletal mass index, SMM: Skeletal muscle mass, DXA: Dual-Energy X-ray Absorptiometry, BMD: bone mineral density, *r_s_*: Spearman correlation coefficient. Bold formatting was used to highlight statistically significant *p*-values (*p* < 0.05).

**Table 3:** Comparison of bioelectrical impedance analysis (BIA) results among the osteoporosis groups.

Parameter	BisP (*n* = 50)	Dmab (*n* = 50)	Osteo (*n* = 50)	*p* ^†^
ALM (kg)	17.79 ± 0.21	18.14 ± 0.22	18.10 ± 0.22	0.455
SMM (kg)	23.65 ± 0.29	24.54 ± 0.29	23.61 ± 0.29	**0.045**
ASM (kg)	16.86 ± 0.20	17.10 ± 0.21	17.13 ± 0.21	0.588
ASMI (kg/m^2^)	7.26 ± 0.05	7.43 ± 0.05	7.34 ± 0.05	0.075

Data are presented as mean ± standard error (SE). ^†^: One-way analysis of covariance. Bold formatting was used to highlight statistically significant *p*-values (*p* < 0.05). Confounding factors: Age, BMI, Charlson Comorbidity Index, serum Vitamin D levels and history of pathological fractures. ALM: Appendicular lean mass, ASM: Appendicular skeletal mass, ASMI: Appendicular skeletal mass index, SMM: Skeletal muscle mass, BisP: patients undergoing bisphosphonate treatment, Dmab: patients undergoing denosumab treatment, Osteo: patients who had not received any treatment for osteoporosis.

**Table 4:** Classifications of sarcopenia according to EWGSOP2 criteria in the osteoporosis groups.

Sarcopenia Classification	BisP (*n* = 50)	Dmab (*n* = 50)	Osteo (*n* = 50)	Control (*n* = 50)
Probable, n (%)	14 (28.0) *^a^*	4 (8.0) *^b^*	14 (28.0) *^a^*	3 (6.0)
Confirmed, n (%)	1 (2.0) *^a^*	0 (0.0) *^a^*	2 (4.0) *^a^*	0 (0.0)
Severe, n (%)	1 (2.0) *^a^*	3 (6.0) *^a^*	0 (0.0) *^a^*	0 (0.0)
No Sarcopenia, n (%)	34 (68.0) *^a^*	43 (86.0) *^a^*	34 (68.0) *^a^*	47 (94.0)

Data are presented as n (%), Fisher–Freeman–Halton test, *^a^*^,*b*^ indicate differences between groups within each row. There are no statistically significant differences between groups with the same superscripts. BisP: patients undergoing bisphosphonate treatment, Dmab: patients undergoing denosumab treatment, Osteo: patients who had not received any treatment for osteoporosis.

## Data Availability

The data that support the findings of this study are available from the corresponding author upon reasonable request.
